# Urinary tract tuberculosis misdiagnosed and treated as renal carcinoma in the presence of diffuse interstitial nephritis: a case report

**DOI:** 10.1186/s13256-022-03491-8

**Published:** 2022-07-15

**Authors:** Sarya Swed, Mais Barazi, Yamane Chawa, Mahmoud Alhamadeh Alswij, Leena Abdelwahab Alshareef, Sami Bitar, Hazem S. Ghaith, Karam R. Motawea

**Affiliations:** 1 Faculty of Medicine, University of Aleppo, Aleppo, Syria; 2 Department of Internal Medicine, Aleppo University Hospital, Aleppo, Syria; 3 Department of Endocrinology Medicine, Aleppo University Hospital, Aleppo, Syria; 4 Faculty of Medicine and Health Sciences, Red Sea University, Port Sudan, Sudan; 5 Faculty of Medicine, Al-Azhar University, Cairo, Egypt; 6 Faculty of Medicine, Alexandria University, Alexandria, Egypt

**Keywords:** Urinary tract tuberculosis, Pseudotumoral renal tuberculosis, Renal carcinoma

## Abstract

**Background:**

Urinary tract tuberculosis (UTTB) is a common form of extrapulmonary tuberculosis (TB) which can infrequently present as renal carcinoma, leading to serious errors in the diagnosis and treatment of UTTB.

**Case presentation:**

A 76-year-old Syrian man presented with gross hematuria as the main symptom. A urinary endoscopic examination and pelvic multi-slice computed tomography imaging increased the suspicion of a speared renal mass in the right urinary tract. The patient was treated for renal cancer. After nephrectomy and ureterctomy, the histopathology of the resected mass confirmed the diagnosis of UTTB and interstitial nephritis.

**Conclusion:**

This case should serve to increase the attention of clinicians to perform an accurate diagnosis step by step. This is especially important if they have a patient similar to the case described here who presents with a renal mass, to avoid serious results such as the loss of an essential organ system.

## Background

The bacillus* Mycobacterium tuberculosis* causes tuberculosis (TB). About one quarter of the world's population is infected with this bacillus, and it is the leading cause of death due to a single infectious agent and among the top ten causes of death worldwide [1]. There are two types of TB: pulmonary TB, which typically affects the lung, and extrapulmonary TB, which might affect any other organ in the body. Urogenital TB is the third most common form of extrapulmonary TB after lymph node involvement and tuberculous pleural effusion [2]. Urogenital TB usually remains undiagnosed for years because it is a clinically silent disorder. It occurs with a peak incidence in the 20- to 40-year age group and has male:female ratio of 2:1.

The typical classical presentation of urinary tract TB (UTTB) involves the whole urinary collecting system (including renal pelvis, calyces, ureters, and bladder). However, kidney parenchymal lesions are less common, including interstitial nephritis and glomerulonephritis [3]. An atypical manifestation is a pseudotumor, which in extremely rare cases is diagnosed and managed as renal cell carcinoma (RCC). The clinical manifestations of a pseudotumor are often nonspecific symptoms, such as pyuria and microscopic hematuria, as well as symptoms related to the bladder, including dysuria, urgency, nocturia, gross hematuria, low back pain, fever, and weight loss. Patients with advanced disease may have end-stage kidney disease and, rarely, refractory hypertension [4]. The diagnosis of urogenital TB depends on the results of urine examination, radiographic imaging studies, tuberculin skin test, interferon-gamma release assay, and histopathology. Depending on the findings of these diagnostic measures, management with anti-TB drugs is usually the treatment choice, but surgery may be the preferred choice in some cases.

Here we present an extremely rare case reported in the literature that revealed a significant medical mistake in the diagnosis of urogenital TB as RCC. Analysis of the case shows a combination of diffuse and interstitial TB in the same patient.

## Case report

A 76-year-old Syrian man was admitted to the emergency room with a complaint of frequency dysuria and gross hematuria lasting for 6 h. The medical history included hypertension and diabetes mellitus type 2 with no abnormalities. Other medical, family and psychosocial histories were clear. At presentation, the vital signs were stable, and clinical examination revealed that the other organs were normal. Initial hematology tests revealed elevated white blood cell counts (WBCs) (20,000/μL), hemoglobin (HGB) (11 g/dL), mean corpuscular volume (MCV; 82 fl), and platelet counts (254,000/μL), as well as moderate renal insufficiency (creatinine: 1.9 mg/dL; urea: 130 mg/dL). Abdominal echography revealed severe and mild hydronephrosis in the right and left kidney, respectively, with no renal stones or calculus. However, there was a significant thickness in the bladder wall (diameter: 1.5 cm).

**Fig. 1:**
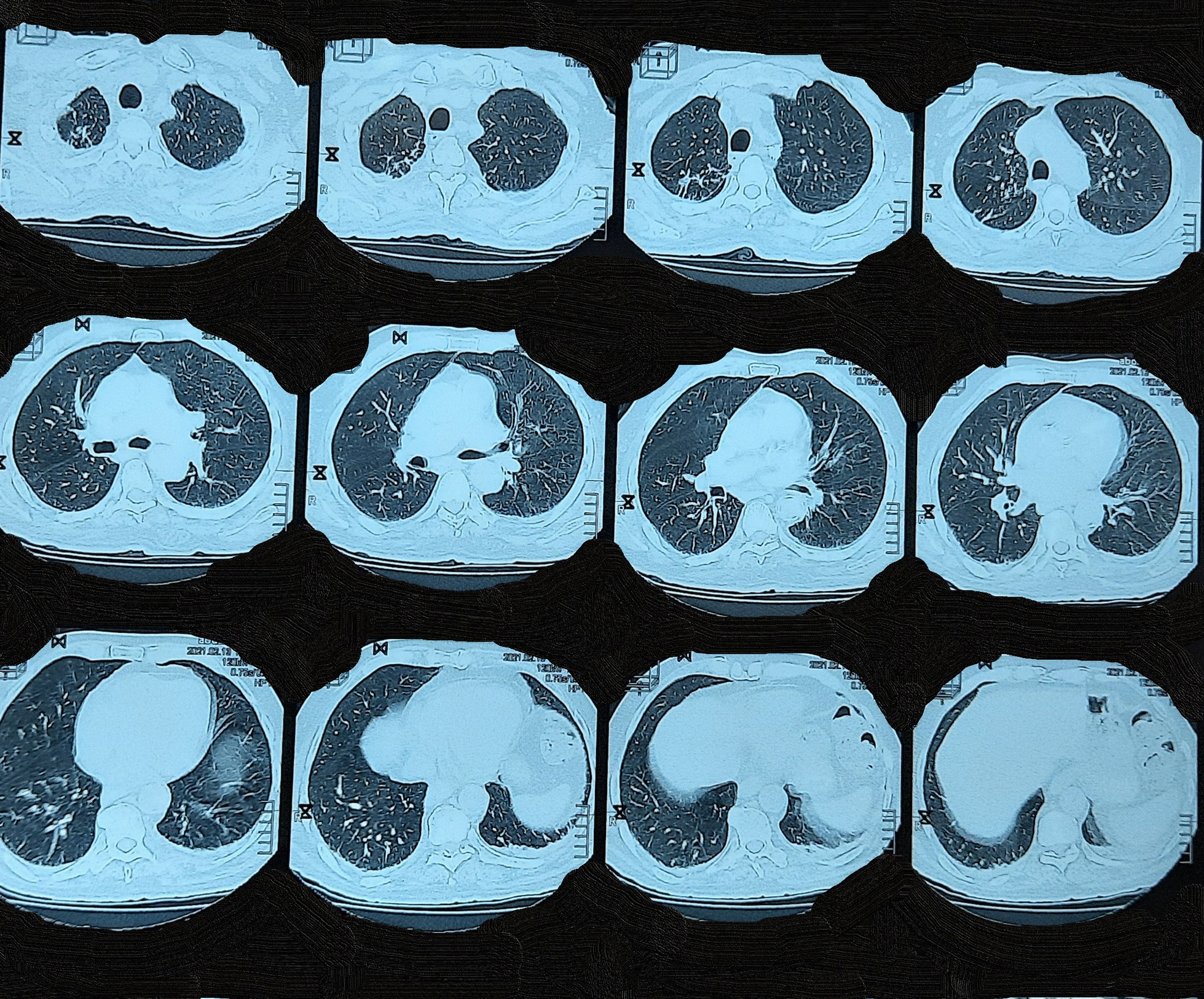
Chest computed tomography revealed only the presence of band-like opacities in the right upper lobe

A cystoscopy was then performed to obtain a biopsy. The histopathology report showed pathological changes, possibly suggesting denuded cystitis, low-grade dysplasia, and severe interstitial cystitis. No invasive tumor was found in the specimen examined. Abdominal and pelvic contrast-enhancing multi-slice computed tomography (CT) revealed the presence of hydronephrosis in the right kidney with a filling defect in the pelvis; this was described as a non-enhancing hypodense area in the pelvis and inferior calyces (Fig. 1). Thoracic CT revealed band-like opacities in the right upper lobe.

**Fig. 2:**
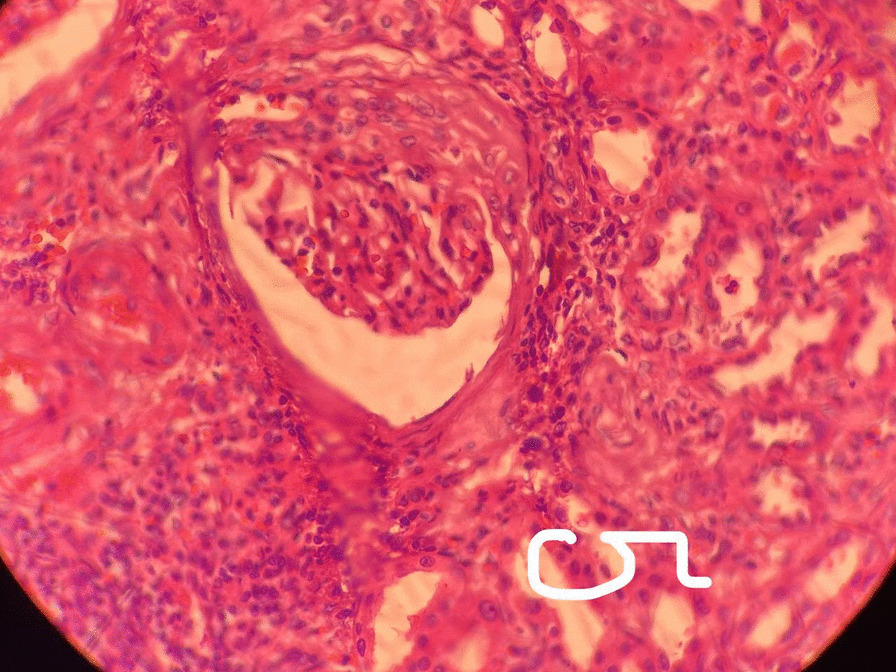
Histopathology examination revealed granulomatous pyelonephritis with necrotizing and caseous centers, raising the suspicion of tuberculosis (First microscopic image)

**Fig. 3:**
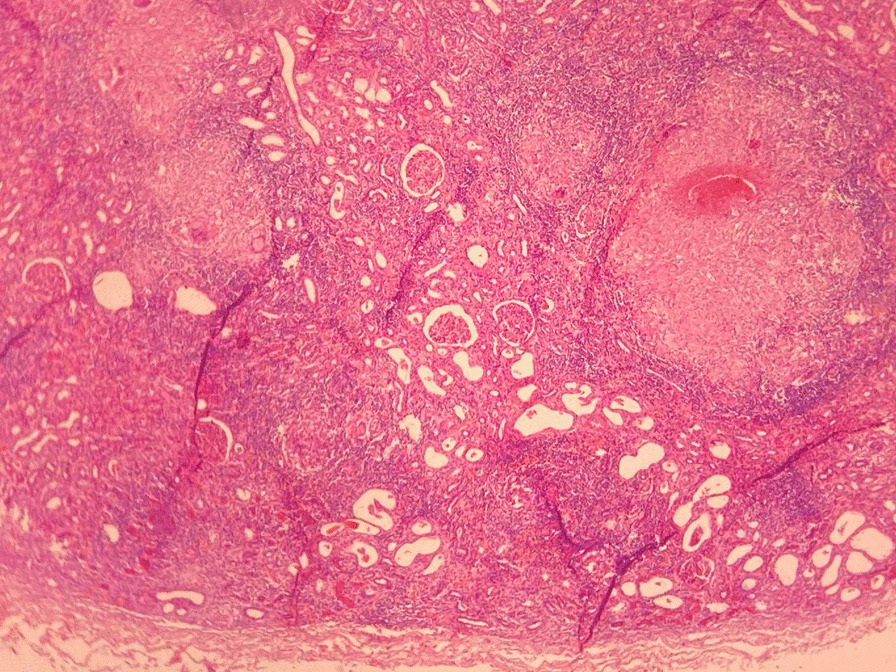
Histopathology examination revealed granulomatous pyelonephritis with necrotizing and caseous centers, raising the suspicion of tuberculosis (Second microscopic image)

A primary diagnosis of RCC was made based on the clinical presentation, the laboratory test results, and the imaging investigations. As a result, the patient underwent an open radical right nephrectomy and ureterectomy. The histopathology examination of specimens showed the existence of granulomatous pyelonephritis and necrotizing and caseous centers, which confirmed the correct diagnosis of urinary tract TB (UTTB). The granulomatous inflammation was extended to most of the ureter, pelvis, and calyces. In addition, patchy infiltrations were present in the renal parenchyma accompanied by focal segmental glomerulosclerosis. The final diagnosis was diffuse interstitial nephritis and UTTB (Figs. 2, 3). Treatment with anti-TB drugs was initiated, with a recommended regular follow-up for 2 years. Unlikely, on day 10 of the follow-up, the patient had an ischemic stroke and died due to stroke complications.

## Discussion

The urogenital system is one of the most common sites of extrapulmonary TB. Urogenital TB is diagnosed in 1.1–1.5% of all TB cases and in 5–6% of cases of extrapulmonary TB [5]. Any portion of the lower urogenital tract can be involved secondarily through antegrade infection from the infected urine. Genital TB is typically a disease of young and middle-aged adults. Most patients present with local symptoms, such as frequent voiding, dysuria, pyuria presenting as lower back, flank, or abdominal pain [6], and microscopic or macroscopic hematuria. Infrequently, a renal mass mimicks RCC [7]. Thus, a high degree of suspicion is warranted with patients from countries with a high prevalence of TB.

The differential diagnosis of enhancing renal mass is an abscess, primary renal tumor-like RCC, and lymphoma or secondary metastasis [8]. Most of the cases reported in the literature on the pseudotumor presentation of urogenital TB involved the presence of a unilateral mass mimicking RCC, and the diagnosis was always made after radical nephrectomy [8], as in the case presented here. Patients with this form of renal mass, known as the pseudotumoral type, present variably sized but well-defined parenchymal nodules on cross-sectional images. The lesion may simulate a renal hydatid cyst or pseudotumoral xanthogranulomatous pyelonephritis. However, genitourinary TB may present as well-defined parenchymal nodules of variable sizes, sparing the collecting system in what is known as the pseudotumoral type. With clinical and radiological findings suggestive of RCC, the patient undergoes surgical removal of the involved kidney, with the subsequent histopathology examination unexpectedly establishing the diagnosis of TB [8]. In addition to the above, TB-associated diffuse tubulointerstitial nephritis is rare and needs rapid recognition and early treatment. Kidney biopsy should be performed in patients with TB and renal disease to ensure the diagnosis of renal involvement of active TB and establish the correct treatment. The mainstay of treatment for this disease is a regimen of anti-TB therapy consisting of isoniazid and rifampicin for 5 months, supplemented in the first 2 months with pyrazinamide and ethambutol.

In the case described here, a 76-year-old man presented with complaints of frequency dysuria and gross hematuria. Laboratory tests showed elevated creatinine and urea levels. Based on multi-slice CT imaging of the abdomen and pelvis, we diagnosed RCC as the diffuse mass in the right urinary tract, so the treatment decided upon was open right nephrectomy and urethrotomy. However, based on the histopathology of the specimens taken after the open surgery, the diagnosis was diffuse interstitial nephritis and UTTT. This case is distinctive and rare due to the presence of two forms of diffuse and interstitial TB. This patient was treated with anti-TB therapy consisting of 6 months of isoniazid and rifampicin, supplemented with pyrazinamide and ethambutol in the first 2 months.

## In conclusion

Diagnosing UTTB as a malignant tumor and treating it with surgical removal is a reality due to TB appearing in the form of a mass. In addition, the detection of two types of TB, namely, diffuse and interstitial TB, in one case increases the rarity of the case. This case illustrates the care required of the radiologist and pathologist to accurately diagnose patients who present with a kidney mass and ensure that they receive standard anti-TB treatment and follow-up.

## Data Availability

Not applicable.
